# Fine-scale spatial and temporal dynamics of *kdr* haplotypes in *Aedes aegypti* from Mexico

**DOI:** 10.1186/s13071-018-3275-9

**Published:** 2019-01-09

**Authors:** Marissa K. Grossman, Julian Rodriguez, Anuar Medina Barreiro, Audrey Lenhart, Pablo Manrique-Saide, Gonzalo M. Vazquez-Prokopec

**Affiliations:** 1 Department of Entomology, Pennsylvania State University, University Park, PA USA; 2 Department of Environmental Sciences, Emory University, Atlanta, GA USA; 3 Departamento de Zoología, Campus de Ciencias Biológicas y Agropecuarias, Universidad Autónoma de Yucatán, Mérida, Yucatán Mexico; 4 Center for Global Health/Division of Parasitic Diseases and Malaria/Entomology Branch, U.S. Centers for Disease Control and Prevention, Atlanta, GA USA

**Keywords:** *Aedes aegypti*, *kdr*, Pyrethroid, Insecticide resistance

## Abstract

**Background:**

As resistance to insecticides increases in disease vectors, it has become exceedingly important to monitor populations for susceptibility. Most studies of field populations of *Aedes aegypti* have largely characterized resistance patterns at the spatial scale of the city or country, which may not be completely informative given that insecticide application occurs at the scale of the house or city block. Phenotypic resistance to pyrethroids dominates in *Ae. aegypti*, and it has been partially explained by mutations in the voltage-gated sodium channel gene. Here, we assess community-level patterns of four knockdown resistance (*kdr*) haplotypes (C1534/I1016, F1534/I1016, C1534/V1016 and F1534/V1016) in *Ae. aegypti* in 24 randomly chosen city blocks from a city in Yucatán State, Mexico, during both the dry and wet season and over two years.

**Results:**

Three of the four haplotypes, C1534/I1016, C1534/V1016 and F1534/V1016 were heterogeneous between city blocks at all four sampling time points, and the double mutant haplotype, C1534/I1016, showed a significant increase following the wet season. The F1534/I1016 haplotype was rarely detected, similar to other studies. However, when haplotype frequencies were aggregated to a coarser spatial scale, the differences in space and time were obscured.

**Conclusions:**

Our results provide empirical evidence that the selection of *kdr* alleles is occurring at fine spatial scales, indicating that future studies should include this scale to better understand evolutionary processes of resistance in natural populations.

**Electronic supplementary material:**

The online version of this article (10.1186/s13071-018-3275-9) contains supplementary material, which is available to authorized users.

## Background

The recent introduction of Zika and chikungunya into the Americas, along with the global persistence of dengue, has made *Aedes aeygpti* one of the most important disease vectors worldwide [1]. *Aedes aegypti* are highly anthropophilic mosquitoes that live in close association with humans, primarily in urban areas. They live and breed in and around houses, with immature stages developing in a wide variety of peridomestic water-holding containers, making them very difficult to control [2]. *Aedes aegypti* control programs employ a combination of methods including ultra-low volume spraying (ULV), indoor space spraying (ISS), indoor residual spraying (IRS), source reduction, and larviciding [3–5]. Unfortunately, most vector control measures, which are the only means to prevent most diseases spread by *Ae. aegypti*, have largely failed in controlling disease transmission in regions where dengue is endemic [6, 7].

Reliance on chemical control strategies has led to the development of resistance to many of the insecticides used to control *Ae. aegypti* throughout the world [3, 8]. Historically, the use of pyrethroid insecticides for vector control has been widespread due to their low cost and low mammalian toxicity [9] and consequently pyrethroid resistance has become a major source of treatment failure [7]*.* In *Ae. aegypti*, resistance to pyrethroid insecticides most commonly arises due to increased metabolic activity or structural alterations at the target site of the insecticide [9]. Point mutations in the *para*-orthologous sodium channel gene are associated with resistance to both pyrethroid and organochlorine insecticides in *Ae. aegypti* [10]. Collectively termed “knockdown resistance”, or *kdr*, these mutations reduce the ability of insecticides to bind to the sodium channel [9]. Several *kdr* mutations have been identified in *Ae. aegypti* [3] and two have been previously associated with pyrethroid resistance in *Ae. aegypti* from Mexico, specifically the valine to isoleucine mutation on codon 1016 of the voltage-gated sodium channel gene and a phenylalanine to cysteine mutation on codon 1534 [11–13]. Both the I1016 and C1534 *kdr* mutations are well established in the literature as significantly associated with pyrethroid resistant phenotypes [3, 14–16], and recently it has been suggested that there is co-evolution of the two markers [12], yet their combined impact on phenotype is not clear.

The frequency of *kdr* mutations has been increasing rapidly in time and space throughout the world. For example, in Mexico, the frequency of I1016 increased from < 0.1% in 1996–2000 to 88.3% in 2007–2009 [11]. Brazil experienced a similar increase in both the C1534 and I1016 alleles: in 2002 neither allele was present, in 2006 both were detected at low frequencies, and by 2012 both alleles were widely prevalent [17]. Not only are *kdr* alleles increasing in frequency in locations where they had previously been detected, but they are also being detected in new locations at relatively high frequencies. For example, the first report of *kdr* in Indian *Ae. aegypti* in 2015 detected a frequency of C1534 of up to 0.79 in one location in the country [18]. Similarly, the G1016 and P989 *kdr* alleles were recently widely detected above 80% frequency in Myanmar [19]. Given these increases, it has become important to understand the patterns of resistance associated with *kdr* and their persistence in natural populations.

Previous studies of *kdr* in field populations of *Ae. aegypti* have largely extrapolated regional patterns of *kdr* frequencies from sampling relatively few locations in a country [17, 18, 20], or they offer a snapshot through cross-sectional studies that report *kdr* frequencies taken at a single time point [19, 21, 22]. These sampling schemes, which pool specimens from multiple breeding sites, would be appropriate if *Ae. aegypti* populations are panmictic at coarse spatial scales (e.g. province or country), as previous research has suggested [23], or if sampling occurred within the limited flight range of *Ae. aegypti* (approximately 50–150 m [24]). However, the heterogeneity of insecticide selection pressures may render this assumption invalid. One hypothesis is that the use of ULV applications of insecticides to control *Ae. aegypti* would lead to homogeneous distributions of resistance within larger geographic areas (e.g. neighborhoods). Conversely, the use of ISS or IRS for the control of *Ae. aegypti* may lead to a more heterogeneous pattern, driven by the extent and frequency of highly focused insecticide applications [25]. Given the reactive nature of dengue control, and the potential for strong variability in insecticide selection pressure in space and time, there is a need to understand the spatial scale of insecticide resistance dynamics.

A recent study by Deming et al. [13] found significant heterogeneity in *kdr* frequencies between interconnected towns in Yucatán State, Mexico, with some towns displaying heterogeneity between city blocks. Building on this research, a comprehensive sampling of *Ae. aegypti* was conducted in one of the study towns over a two year period, aiming to answer two questions: (i) are *kdr* frequencies heterogeneous at the spatial scale of city blocks, or are they more accurately assessed at the neighborhood level, and (ii) to what extent can *kdr* frequencies vary over a relatively short timeframe, namely between the wet and dry season within a year? Understanding the spatial and temporal dynamics of *kdr* frequencies is not only important for monitoring insecticide susceptibility, but also for designing interventions to mitigate and manage resistance while controlling populations effectively.

## Methods

### Study area

**Fig. 1:**
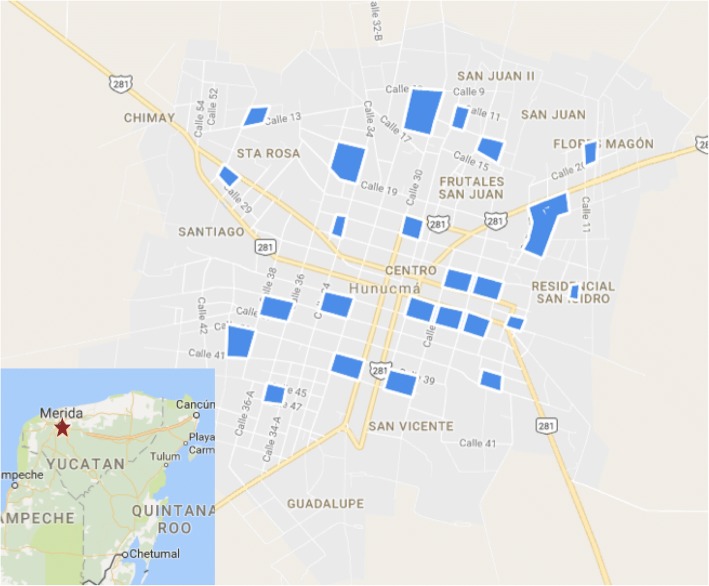
Study area in Hunucmá, Yucatán, Mexico. The 24 study blocks are shaded in blue and the four sampling sectors are defined by the two main roads. The inset shows the location of Hunucmá (red star) in the Yucatán Peninsula. Source: Google maps

This study was conducted in Hunucmá (population approximately 25,000), a small satellite town of the city of Merida, the capital of Yucatán State, Mexico (Fig. 1). Hunucmá is endemic for dengue virus transmission, and there were reports of both chikungunya and Zika transmission in 2016 (Secretary of Health, Yucatán).

Mexico’s national vector control program utilizes ULV, ISS and larviciding to control *Ae. aegypti*. In the year 2000, the program began using permethrin-based products, but began transitioning to non-permethrin products in 2009 [11, 26]. At the time of the study in Hunucmá, the insecticides malathion, chlorpyrifos, bendiocarb and deltamethrin were used for adult *Ae. aegypti* control (Secretary of Health, Yucatán). Previous research indicated that in Hunucmá in 2013, the *Ae. aegypti* population was resistant to deltamethrin and the frequency of the C1534 *kdr* allele was 0.568 and I1016 was 0.425 [13].

### Insecticide use data

The Yucatán’s Secretary of Health provided information from their Entomological Surveillance and Vector Control Database [27] about the *Ae. aegypti* control efforts in Hunucmá during the duration of the study. These data included the types of insecticide, mode of application (ULV, ISS, or larvicide), date and location of application at the city block level. Insecticide-based interventions were typically conducted reactively in response to reported dengue or chikungunya cases.

### Entomological surveys

**Fig. 2:**
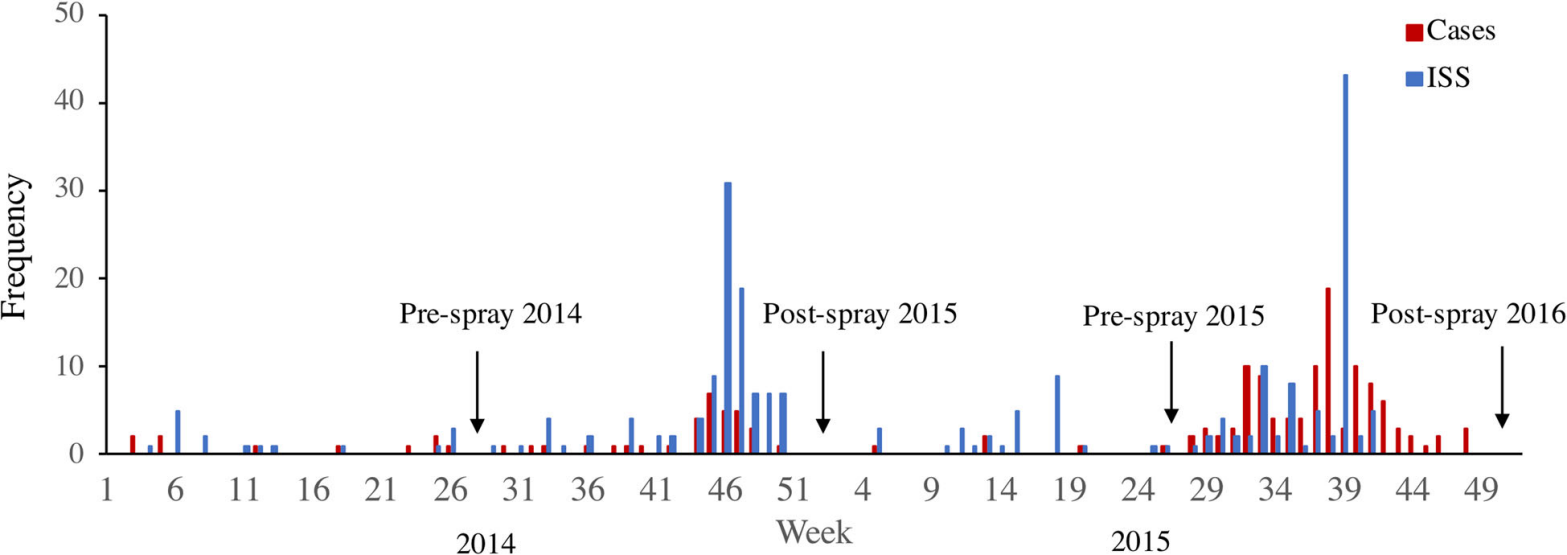
Dengue and chikungunya cases in Hunucmá during 2014 and 2015 (Secretary of Health, Yucatán). Entomological sampling points are marked with arrows

We conducted a longitudinal entomological survey twice per year from June 2014 to January 2016 at the beginning of the wet season (June-July; before the peak dengue transmission season) and at the beginning of the dry season (January; after the peak dengue transmission season) (Fig. 2). Hunucmá has two main roads that run northeast-southwest and northwest-southeast, dividing the city into four geographical areas. *Aedes aegypti* have been shown to be highly clustered in space [28] with dispersal limited by the urban landscape, such as roads [29]. As such, these four areas were chosen as the course-scale sampling units, which we define as sectors (Fig. 1). Within the three smaller sectors, five blocks were selected at random to sample for fine-scale dynamics, and in the larger sector, nine blocks were selected at random, totaling 24 blocks. Power calculations for the test of two proportions with alpha = 0.025 (two-sided hypothesis) and a beta = 0.2 (80% power) indicated that approximately 30 mosquitoes per block were needed to detect a 25% difference in allele frequencies per block. Previous entomological surveys estimated 3–5 adult female *Ae. aegypti* could be collected per house, so we sampled at least 10 houses per block, or enough houses until we obtained a sample of at least 30 individuals.

We used a Prokopack aspirator [30] to collect adult mosquitoes from each house for 10 minutes or until we sampled from every available room. All collected mosquitoes were transported in plastic cups to the laboratory at the Universidad Autonoma de Yucatán in Merida, where they were placed at -20 °C for euthanization. We identified each mosquito to the species level and sex, and *Ae. aegypti* were stored in RNAlater*®* Stabilization Solution (Thermo Fisher Scientific, Waltham, MA, USA) for future *kdr* genotyping.

During the dry season collections in January of each year, there were low numbers of adult mosquitoes collected inside homes. To augment sampling efforts and supplement Prokopack collections, we placed oviposition traps (ovitraps) outside four houses per block (one on each side of the block) for up to 4 weeks to collect *Ae. aegypti* eggs. The eggs were then aggregated by block and reared in the insectary at the Universidad Autonoma de Yucatán. Thirty-five to fifty adults per block emerging from the eggs collected from the ovitraps were selected at random for subsequent *kdr* genotyping. Adults from ovitraps were only used to complement block collections with less than 30 adults collected by Prokopacks. Our strategy of using four traps per block over a period of 4 weeks aimed at minimizing bias typical of ovitrap collections (i.e. sampling related individuals at a rate higher than what we would collect indoors with Prokopacks). One city block had enough mosquitoes to compare adult and ovitrap data and yielded no significant differences in the frequency of *kdr* mutations in both samples (for Prokopack, the frequency of C1534 was 18/30 and with the ovitrap it was 20/26; *χ*^2^ = 1.13, *df* = 1, *P* = 0.2867; for I1016 it was 14/30 with the Prokopack and 10/26 with the ovitrap; *χ*^2^ = 0.121, *df* = 1, *P* = 0.7278).

### Molecular assays

Both male and female mosquitoes collected from blocks were used for genotyping since they both contribute to the genetic pool of the next generation. Individual mosquitoes were placed in individual wells of a 96-well plate in a 50 μl solution containing 5 μl of Taq 10× buffer (containing 500 mM KCl, 100 mM tris HCl, 15 mM MgCl_2_ and 1% Triton X-100) and 45 μl of sterile ddH_2_O and heated in an Eppendorf Mastercycler© Pro thermocycler at 95 °C for 15 min. Allele-specific real time PCR was conducted using a BioRad© CFX96 real-time PCR machine (Hercules, CA, USA) based on the protocols and primer sequences described by Saavedra-Rodriguez et al. [14] for position 1016 and Yanola et al. [15] for position 1534. Reaction conditions were as follows: for locus 1016, each 20 μL reaction consisted of 8 μl of PerfeCTa® SYBR® Green FastMix (Quanta Biosciences, Gaithersburg, MD, USA), 8.86 μl of ddH_2_O, 0.34 μl of 10 μM Val1016 forward primer, 0.4 μl of the 10 μM Iso1016 forward primer, 0.4 μl of the 10 μM Iso1016 reverse primer and 2 μl of template DNA; for locus 1534, each 20 μl reaction consisted of 9 μl of PerfeCTa® SYBR® Green FastMix (Quanta Biosciences), 7.15 μl of ddH_2_O, 0.65 μl of 10 μM Cys1534 forward primer, 0.60 μl of the 10 μM Phe1534 forward primer, 0.60 μl of the 10 μM Cys1534 reverse primer and 2 μl of template DNA. Melting curve analyses determined the genotypes of the mosquito at each codon [14, 15]. Each PCR plate included three control *Ae. aegypti* mosquitoes of known genotypes at the two *kdr* loci: a homozygous wild-type individual, a heterozygous individual and a homozygous mutant individual (separate controls were used for each mutation).

### Data analysis

The F1534C and V1016I mutations are located close together on the chromosome, so we tested for linkage disequilibrium between the two markers by using the equations outlined in Gillespie [31] to calculate the coefficient D, *r*^2^, and the Chi-square statistic (*χ*^2^) with one degree of freedom. The mutations were in linkage disequilibrium for all time points (First time point: *D* = 0.137, *r*^2^ = 0.31, *χ*^2^ = 541.9, *P* < 0.0001), so we estimated haplotype frequencies for each block and sector at each time point. Full methods for haplotype calculations are found in Additional file 1.

Haplotype frequencies, except for F1534/I1016 which was rarely detected, were mapped using QGIS 2.18 (QGIS Development Team, 2016) at both the block and sector level for each sampling time point. Sampling time points in June-July were characterized as the wet season and time points in January were defined as the dry season.

We used a multiple proportions test with a Chi-square distribution to test if at least one city block or sector had significantly different haplotype frequencies than the others for each time point. The same test was used to see which blocks or sectors had different haplotype frequencies at least one point in time. The presence of spatial autocorrelation between block-level allele frequencies was assessed with the Moran’s I statistic using an inverse distance weighted scheme in the R package ape [32]. Moran’s I is similar to Pearson’s r correlation, yet it tests correlation of values in space. To test for an overall effect of time on block-level allele frequencies, we used a linear mixed model with time point as the predictor and block nested within sector as the random intercept using the R package *nlme* [33]. Additionally, the effect of season on block-level allele frequencies was analyzed by combining the data from the two dry and two wet season collections. For this analysis, we used a linear mixed model with block as a random intercept and season as fixed effect. Lastly, we used a linear mixed model with block as a random intercept to assess the relationship between the mean number of adult mosquitoes collected in the houses per block and the allele frequencies per block. Only wet season data were used for this analysis due to the limited number of mosquitoes collected inside houses during the dry season.

## Results

### Insecticide application

**Fig. 3:**
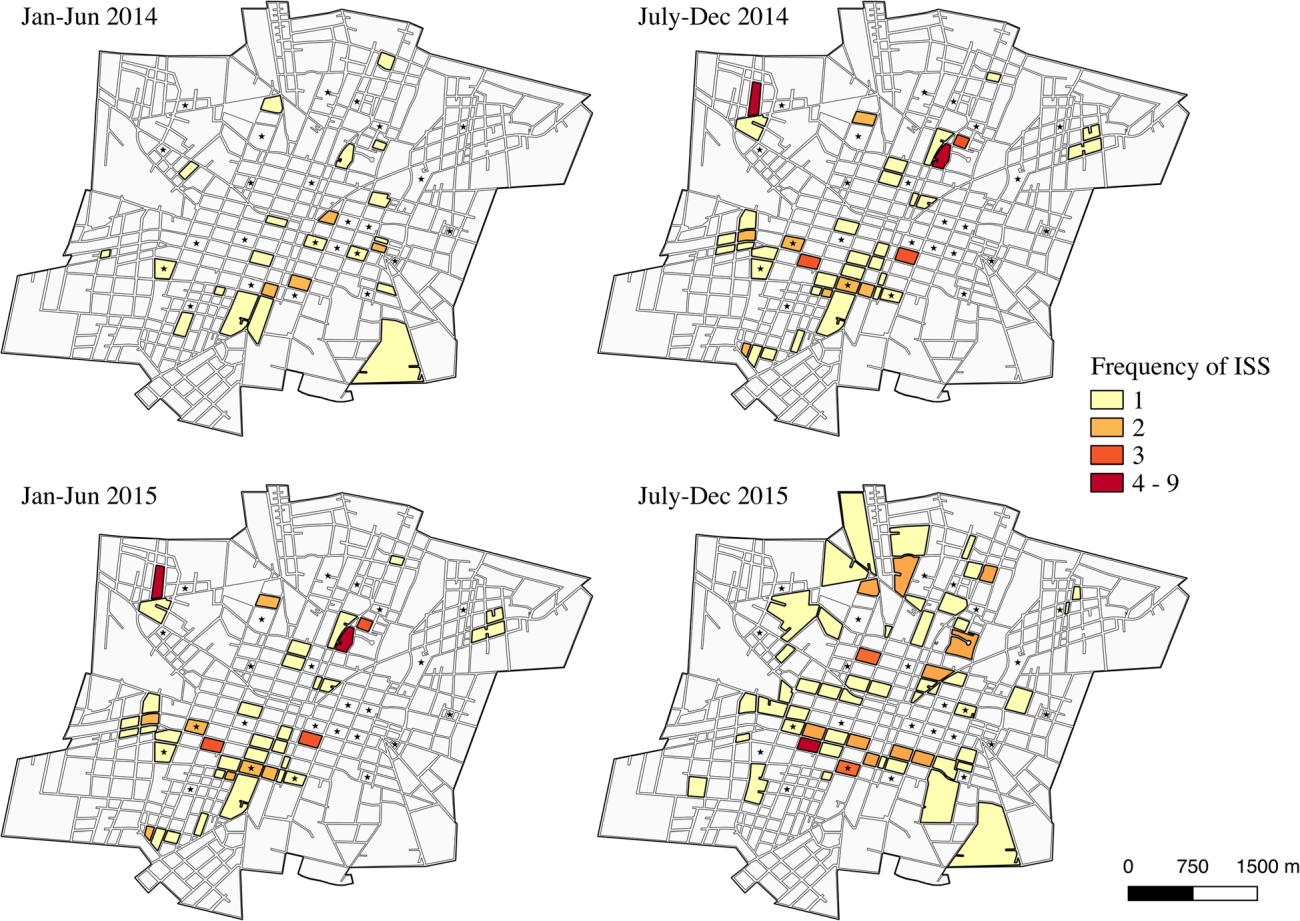
Indoor space spraying with deltamethrin. January to June represents the dry season, and the wet season occurs from August to November. Study blocks are marked with an asterisk

Insecticide applications were highly heterogeneous in space. In 2014 and 2015, the insecticides malathion and chlorpyrifos were used for ULV spraying in the study area. Both deltamethrin (a pyrethroid insecticide) and bendiocarb (a carbamate) were used for ISS, although only 6 out of the 24 study blocks (25%) received ISS at any point during the 2-year study period, so we could not assess any relationship between ISS and *kdr* frequencies (Fig. 3).

### Entomological surveys

A total of 571 houses were sampled for adult mosquitoes from June 2014 to Feb 2016, yielding a total of 3570 *Ae. aegypti*, 63% of which were female (Table 1). The mean number of *Ae. aegypti* adult mosquitoes collected per house was 3.5, so data were not analyzed at the house level, only at the block and sector level to ensure sufficient sample size. The mean (± SD) number of indoor resting adult *Ae. aegypti* per block for each of the four time points was 39.5 ± 22.1 in the wet season in 2014, 9.1 ± 10.9 in the dry season of 2015, 17.1 ± 15.6 in the wet season of 2015, and 2.2 ± 3.1 in the dry season of 2016. If 30 individuals were not caught in a block at a given time point, adults reared from eggs collected on that block were used for genotyping as previously described. All dry season samples consisted of both adults captured inside houses and adults reared from eggs collected on the blocks. Wet season collections were only mosquitoes captured inside houses. The total numbers of adult *Ae. aegypti* per block used for genotyping along with their *kdr* frequencies are presented in Additional file 2: Table S1 and Additional file 3: Table S2, with *kdr* haplotypes in Additional file 4: Table S3.

**Table 1:** Summary statistics for all *Ae. aegypti* collected and analyzed from Hunucmá, Mexico

Time point	No. of females collected	No. of males collected	Total no. collected	No. of C1534 genotyped	No. of I1016 genotyped
Wet 2014	801	360	1161	1070	882
Dry 2015	579	354	933	764	743
Wet 2015	617	334	951	708	665
Dry 2016	345	324	669	660	652
Total	2342	1372	3714	3202	2942

### *kdr* haplotype frequencies in space

**Fig. 4:**
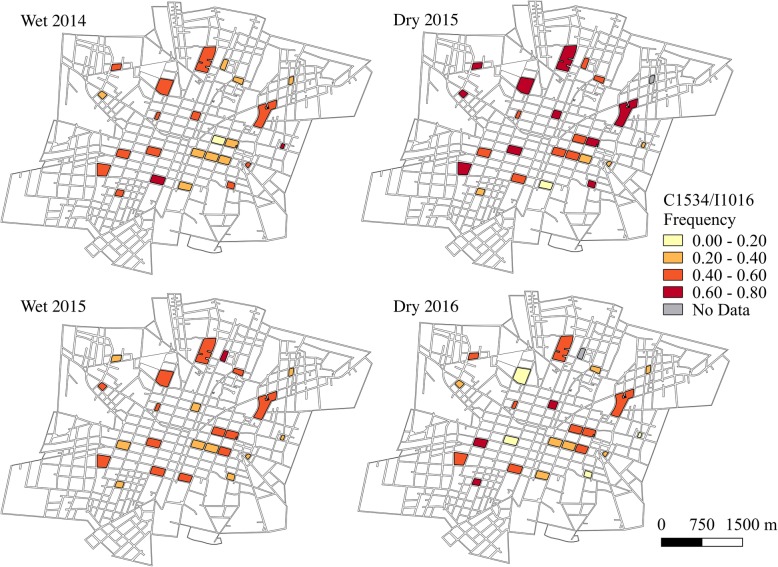
C1534/I1016 haplotype frequencies over time at the block level. Absence of data at a time point indicates that no *Ae. aegypti* were collected on that block during that time

**Fig. 5:**
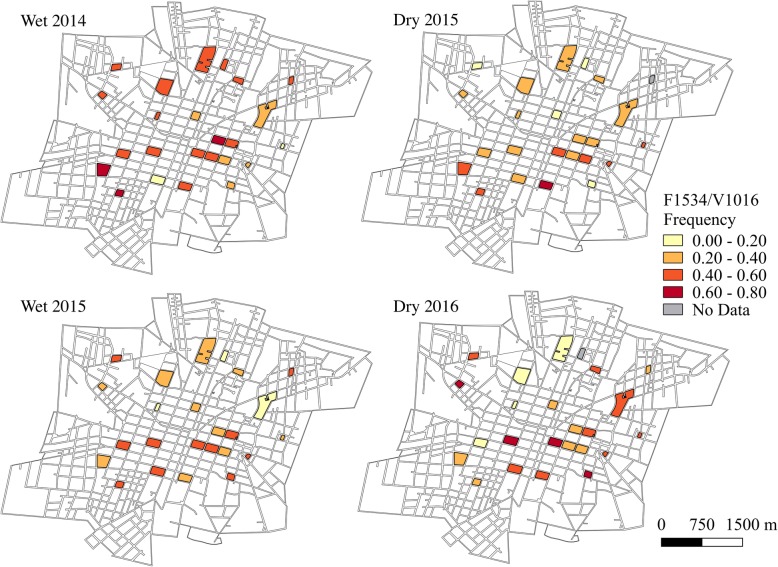
F1534/VI1016 haplotype frequencies over time at the block level. Absence of data at a time point indicates that no *Ae. aegypti* were collected on that block during that time

**Fig. 6:**
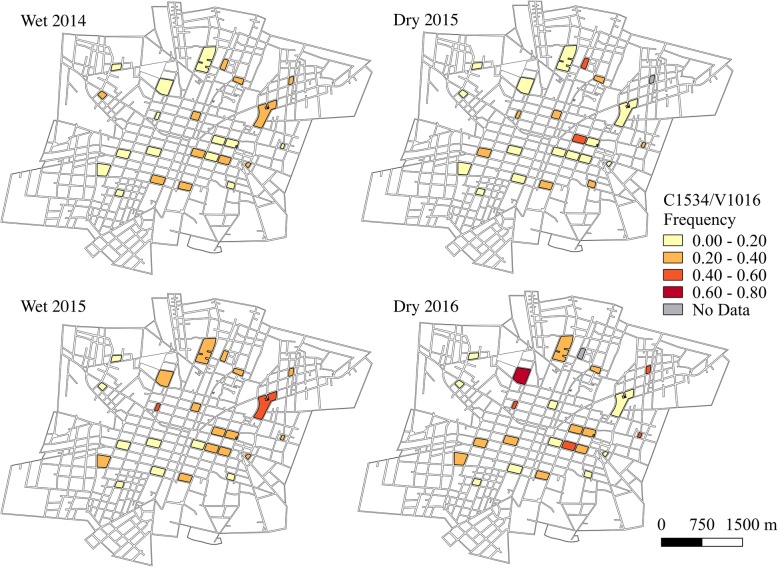
C1534/V1016 haplotype frequencies over time at the block level**.** Absence of data at a time point indicates that no *Ae. aegypti* were collected on that block during that time

The frequencies of the double mutant haplotype, C1534/I1016 (Fig. 4), and the wild-type haplotype, F1534/V1016 (Fig. 5), varied greatly at the block-level throughout the course of the two-year study period. At all time points, the frequencies of C1534/I1016 were significantly different between blocks (wet 2014: *χ*^2^ = 141.2, *df* = 23, *P* < 0.001; dry 2015: *χ*^2^ = 307.6, *df* = 22, *P* < 0.001; wet 2015: *χ*^2^ = 84.1, *df* = 23, *P* < 0.001; dry 2016: *χ*^2^ = 226.4, *df* = 22, *P* < 0.001). Similarly, frequencies of F1534/V1016 showed significant differences between blocks at all time points (wet 2014: *χ*^2^ = 142.9, *df* = 23, *P* < 0.001; dry 2015: *χ*^2^ = 326.63, *df* = 22, *P* < 0.001; wet 2015: *χ*^2^ = 126.3, *df* = 23, *P* < 0.001; dry 2016: *χ*^2^ = 305.6, *df* = 22, *P* < 0.001). The frequency of the C1534/V1016 haplotype remained relatively low throughout time, yet still showed significant differences between blocks at all time points (Fig. 6; wet 2014: *χ*^2^ = 155.2, *df* = 23, *P* < 0.001; dry 2015: *χ*^2^ = 210.7, *df* = 22, *P* < 0.001; wet 2015: *χ*^2^ = 97.3, *df* = 23, *P* < 0.001; dry 2016: *χ*^2^ = 304.5, *df* = 22, *P* < 0.001). The F1534/I1016 haplotype was rarely detected and only reached a frequency above 0.1 in one block during the wet season for 2014 and two blocks during the wet season of 2015. No time points demonstrated statistically significant evidence of spatial autocorrelation (*I* < |0.05|, *P* > 0.05), indicating that *kdr* haplotype frequencies were randomly distributed in space across the sampled city blocks.

**Fig. 7:**
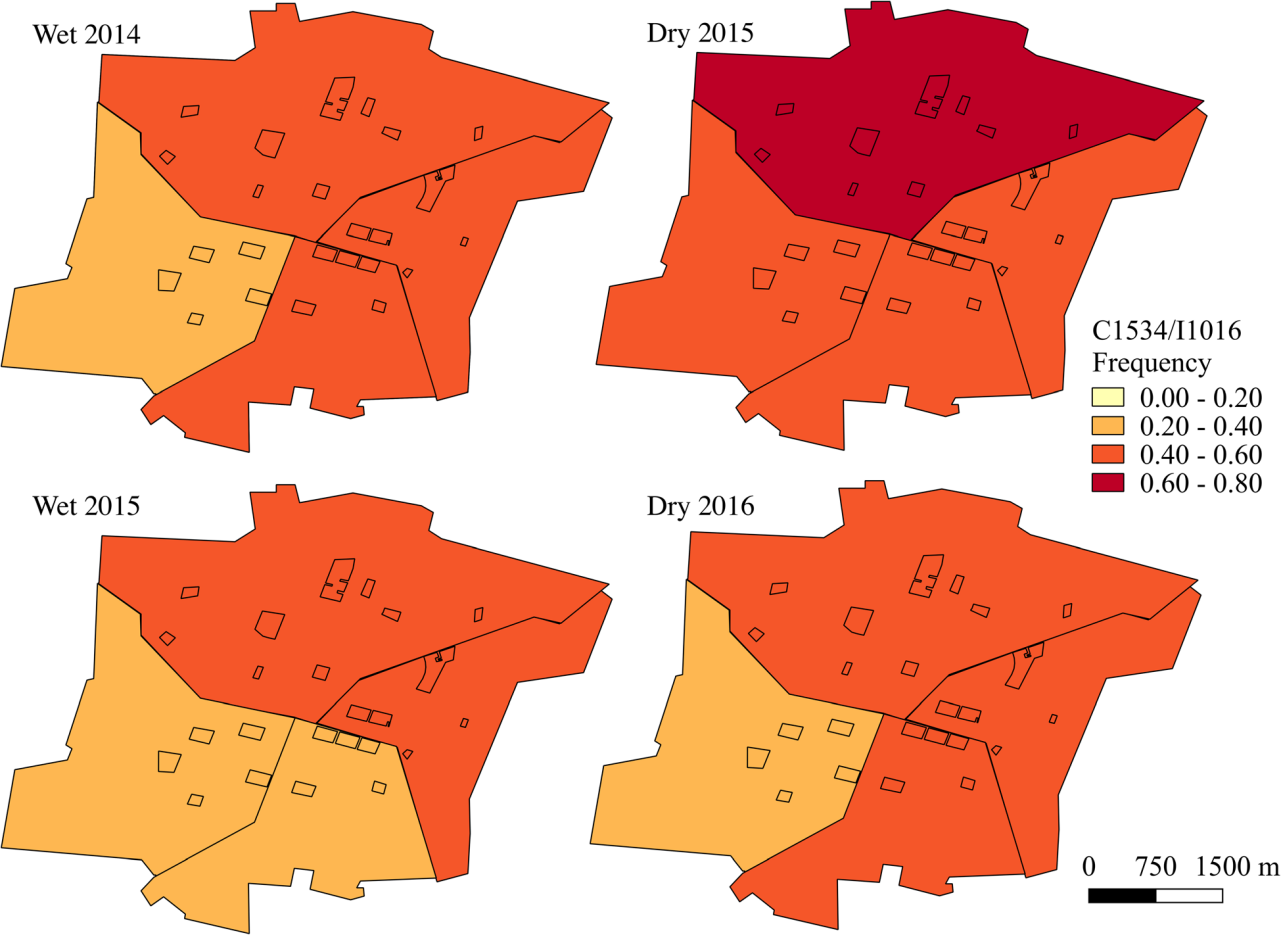
C1534/I1016 frequencies over time at the sector level. Study blocks are outlined in black

**Fig. 8:**
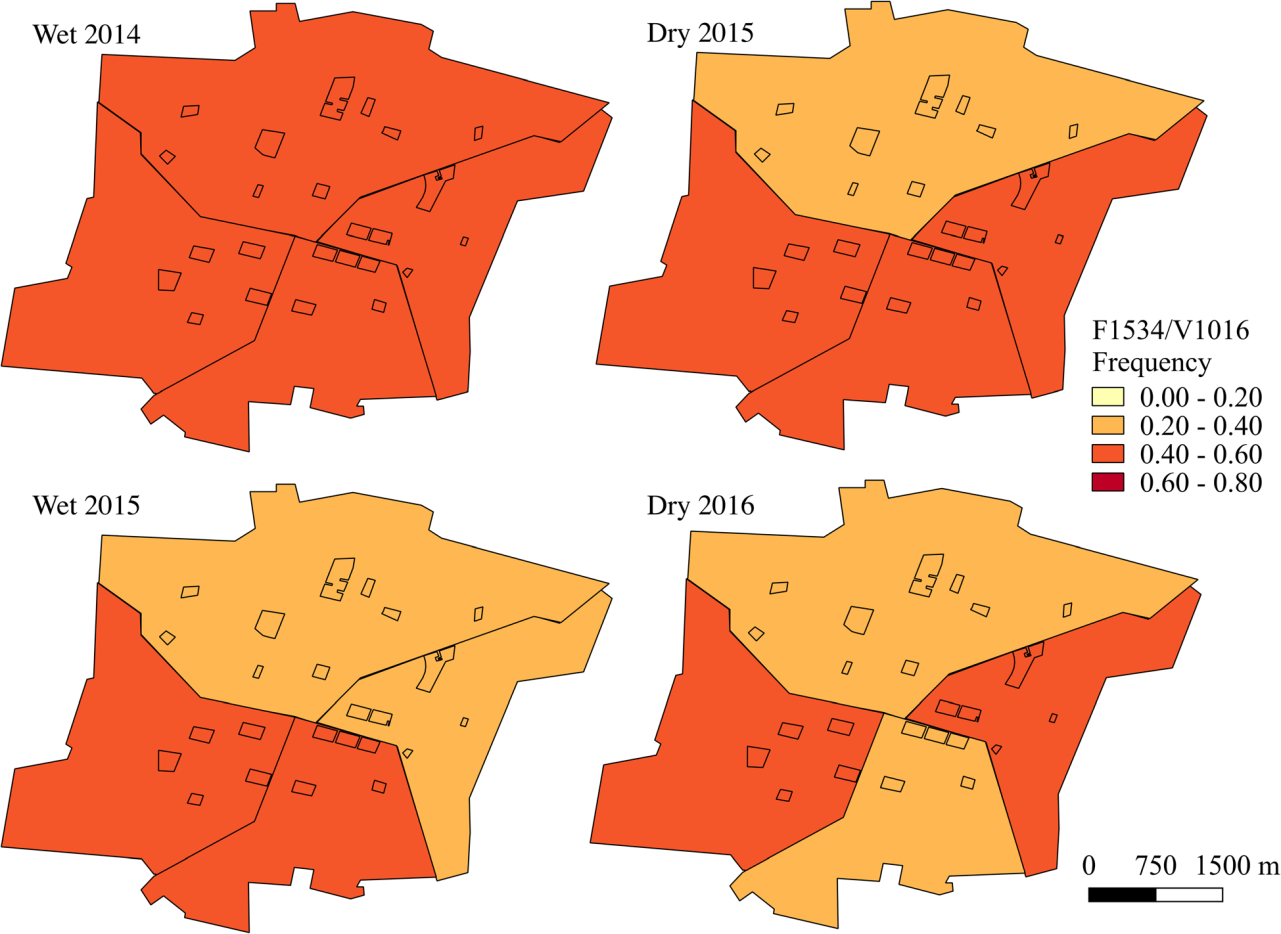
F1534/V1016 frequencies over time at the sector level. Study blocks are outlined in black

**Fig. 9:**
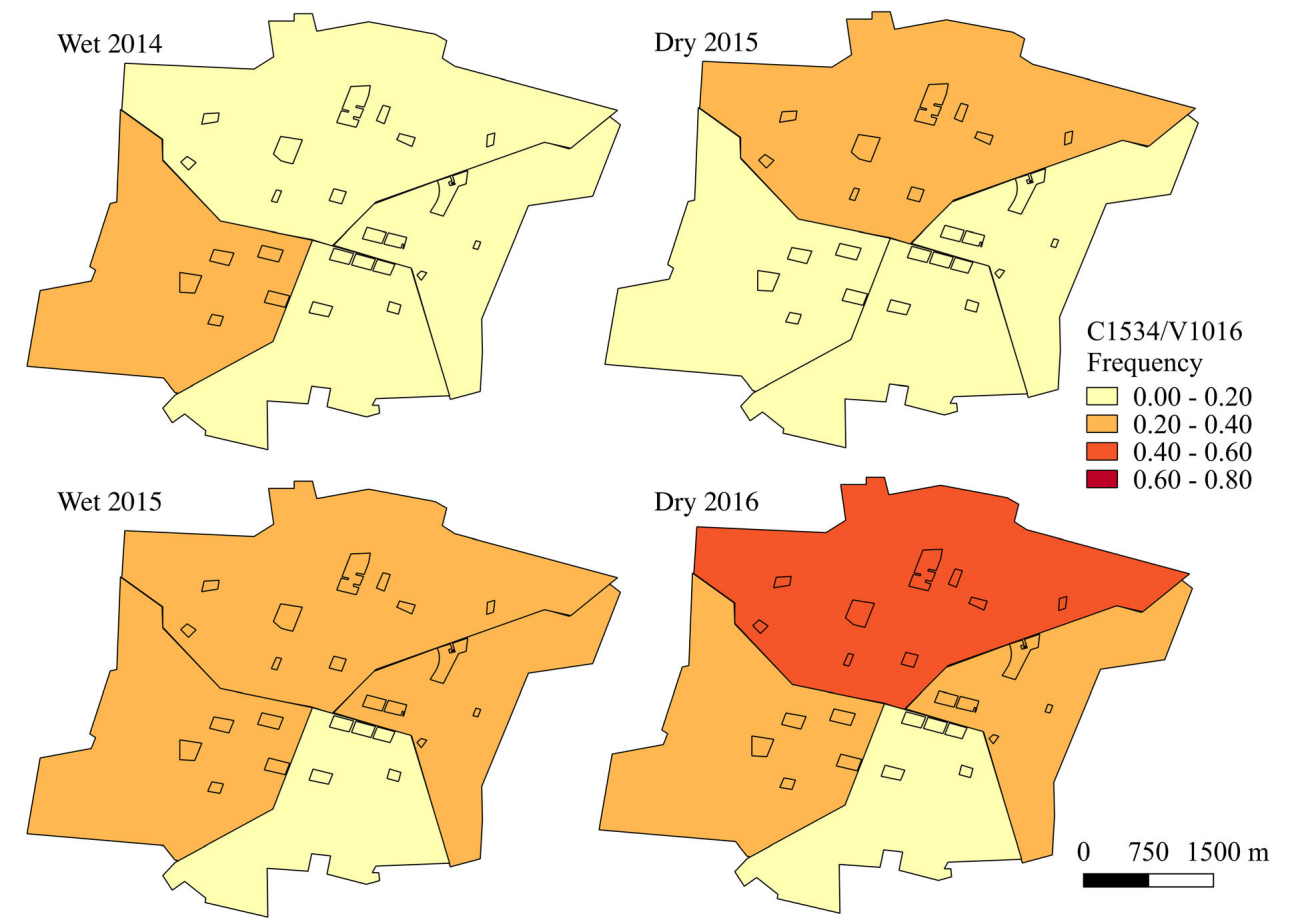
C1534/V1016 frequencies over time at the sector level. Study blocks are outlined in black

When analyzed at the sector level, haplotype frequencies appeared more homogenous (Figs. 7, 8 and 9 and Additional file 5: Table S4). There were only significant differences in the frequencies of the double mutant haplotype, C1534/I1016, between sectors during the dry season time points (dry 2015: *χ*^2^ = 43.0, *df* = 3, *P* < 0.001; dry 2016: *χ*^2^ = 91.5, *df* = 3, *P* < 0.001). However, the frequencies of the wild-type haplotype, F1534/V1016, differed between sectors at all time points (wet 2014: *χ*^2^ = 27.8, *df* = 3, *P* < 0.001; dry 2015: *χ*^2^ = 135.8, *df* = 3, *P* < 0.001; wet 2015: *χ*^2^ = 22.3, *df* = 3, *P* < 0.001; dry 2016: *χ*^2^ = 91.6, *df* = 3, *P* < 0.001), as did the C1534/V1016 haplotype frequencies (wet 2014: *χ*^2^ = 53.1, *df* = 3, *P* < 0.001; dry 2015: *χ*^2^ = 54.0, *df* = 3, *P* < 0.001; wet 2015: *χ*^2^ = 37.3, *df* = 3, *P* < 0.001; dry 2016: *χ*^2^ = 106.1, *df* = 3, *P* < 0.001).

### *kdr* haplotype frequencies over time

The block-level haplotype frequencies also significantly changed over time. Using the first sampling point (wet season 2014) as the reference with a null hypothesis of no change over sampling points, there was a significant increase in the C1534/I1016 haplotype frequency of 0.113 ± 0.04 during the following dry season in 2015 (hierarchical linear mixed-effects model, time effect *t* = 2.67, *P* = 0.009). Accordingly, the wild-type haplotype, F1534/V1016, showed a significant decrease in block-level frequencies of 0.09 ± 0.04 between the wet season of 2014 and the following dry season in 2015 (hierarchical linear mixed-effects model, time effect *t* = -2.11, *P* = 0.038). Aggregating by sector obscured the differences in the wild-type haplotype frequencies over time, showing no significant differences between any of the time points. However, there was still a significant increase of 0.127 ± 0.04 in the frequency of C1534/I1016 between the wet season of 2014 and the dry season of 2015 at the sector level (hierarchical linear mixed-effects model, time effect *t* = 3.10, *P* = 0.0127).

## Discussion

Significant heterogeneity in the frequency of *kdr* haplotypes was detected between city blocks of a dengue endemic town in the Yucatán, suggesting that selection for these haplotypes is occurring at a fine spatial scale. Municipal insecticide application in the study area is highly variable in space and time, creating a patchy mosaic of selection pressures. Theory predicts that in such an environment, if the rate of dispersal or migration between areas is greater than the strength of selection, there will be no local adaptation and the allele with the best average fitness across habitats will increase towards fixation, homogenizing populations [34]. However, if the strength of selection is higher, then local adaptation can occur, with immigration limiting the probability of fixation of an advantageous allele in a given habitat [34]. Population genetics studies have previously shown that *Ae. aegypti* populations in the Yucatán (including Hunucmá) are panmictic [23, 35], suggesting that migration is high and therefore *kdr* frequencies should be similar within towns. Yet, evidence from town-level data [36] and from the present study suggest that local adaptation to pyrethroid insecticides is occurring.

Our data also show temporal heterogeneity in *kdr* haplotype frequencies throughout the year, suggesting rapid selection for these markers. Overall, town blocks showed higher *kdr* haplotypes and allele frequencies during the dry season, which occurs directly following the wet season that coincides with disease transmission. During the wet season, there was an increase in insecticide application to control mosquitoes, subjecting the population to higher selection pressure than during the dry season. The use of household aerosol insecticides, highly prevalent in the Yucatán [37], could have acted as another important selective pressure during the transmission season, though this study unfortunately did not quantify use. However, a previous study from Merida, Mexico found that approximately 87% of households use commercially-available pyrethroid products to control mosquitoes in their homes [37]. Although we were unable to statistically assess the association between insecticide application and *kdr* allele frequencies, it is likely that increased insecticide application during the wet season contributed to the increase in *kdr* haplotypes and allele frequencies that were detected during the dry season.

The relationship between insecticide use and *kdr* frequencies has been well-documented in *Anopheles* spp.; many researchers have detected an increase in *kdr* following deployment of insecticide-treated bednets (ITN) or long-lasting insecticide-treated nets (LLIN), both containing pyrethroids, for malaria prevention [38–40]. For example, Czeher et al. [40] sampled *An. gambiae* in Niger over the course of three years, pre- and post-LLIN intervention, and found that the frequency of the L1014F *kdr* allele was heterogeneous over 14 study sites and increased over time. Similarly, Stump et al. [38] found an increase in the L1014S *kdr* allele in *An. gambiae* in western Kenya following a 15 year period of ITN use, though only in the intervention village and its nearest neighbor (~5 km apart); villages at least 20 km away did not exhibit an increase in *kdr* frequencies. These studies illustrate that insecticide exposures can drive *kdr* frequencies in *Anopheles* spp., and provides justification for further research into these associations in *Ae. aegypti*.

The lower *kdr* haplotype frequencies detected in this study during the early wet season, where the preceding months had low insecticide application, may be indicative of a fitness cost associated with the *kdr* alleles [41–43]. It is encouraging that frequencies of *kdr* haplotypes can be reduced in the field within relatively few mosquito generations, which may facilitate the eventual restoration of insecticide susceptibility if *kdr* is the primary mechanism responsible for insecticide resistance in this population. While phenotypic resistance was not measured in the present study, a previous study found a significant association between the *kdr* alleles and phenotypic resistance to deltamethrin at the same study site [13]. Since *kdr* is not the only mechanism that confers pyrethroid resistance, the role of metabolic resistance mechanisms in this population needs to be studied in greater detail.

## Conclusions

The fine-scale spatial and temporal heterogeneity in the frequency of alleles associated with insecticide resistance has important implications for monitoring susceptibility in field populations. When *kdr* haplotype frequencies were analyzed at the sector level, the differences in time and space were obscured, displaying a more homogeneous pattern. This suggests that important insecticide resistance-related trends may be missed if single sites are assumed to be broadly representative of larger geographies. The marked spatial randomness found in the *kdr* haplotype frequencies at the block level requires a sampling design that selects multiple, random blocks to provide a truly representative picture of *kdr* in an area. Operationally, this would require modifications to existing insecticide resistance surveillance strategies. Additionally, seasonal changes in *kdr* frequencies suggest that cross-sectional studies, or even longitudinal studies that sample at the same time point every year, may not capture evolutionary dynamics. Moreover, how phenotypic resistance is affected by the observed changes in allele frequency needs to be described. Ultimately, understanding the processes underlying the patterns that are observed in the field can inform the improvement of mosquito control strategies and mitigate the negative impacts of the rapid evolution of resistance.

## Additional files

Additional file 1: **Text.** Haplotype frequency calculations. (DOCX 15 kb)

Additional file 2: **Table S1.** Block-level frequencies of C1534 during all four sampling timepoints. Dry season collections were a combination of in-house adult sampling and adults emerging from ovitraps; sample size from each is indicated. Wet season collections only consisted of in-house adult samples. Significant differences are marked with asterisks after correcting for multiple comparisons. (DOCX 21 kb)

Additional file 3: **Table S2.** Block-level frequencies of I1016 during all four sampling timepoints. Dry season collections were a combination of in-house adult sampling and adults emerging from ovitraps; sample size from each is indicated. Wet season collections only consisted of in-house adult samples. Significant differences are marked with asterisks after correcting for multiple comparisons. (DOCX 21 kb)

Additional file 4: **Table S3**. Haplotype frequencies for each block at each sampling timepoint. (DOCX 25 kb)

Additional file 5: **Table S4.** Haplotype frequencies for each sector at each sampling timepoint. (DOCX 14 kb)

## Abbreviation

KDR: Knockdown resistance
