# Supplementary material for: Fine-scale spatial and temporal dynamics of kdr haplotypes in Aedes aegypti from Mexico
Source: Parasit Vectors. 2019 Jan 9;12:20. doi: 10.1186/s13071-018-3275-9 (PMC6327429; doi:10.1186/s13071-018-3275-9)
Supplement: Supplementary file 1 — Text. Haplotype frequency calculations. (DOCX 15 kb) [file 13071_2018_3275_MOESM1_ESM.docx]

**Fine-scale spatial and temporal dynamics of *kdr* haplotypes in *Aedes aegypti* from Mexico**

Marissa K. Grossman, Julian Rodriguez, Anuar Medina Barreiro, Audrey Lenhart, Pablo Manrique-Saide and Gonzalo M. Vazquez-Prokopec

**Additional file 1. Text. Haplotype frequency calculations**

We calculated allele frequencies at the 1534 and 1016 *kdr* loci for each block and then each sector at every sampling timepoint. We assigned 1534 locus *A*, defining $p_{A1}$ as the frequency of F1534 (the wildtype allele), and 1016 was assigned locus *B*, with $p_{B1}$ as the frequency of V1016 (the wildtype allele).

Because these loci are physically close on the chromosome, we calculated linkage disequilibrium between them at each timepoint for each block. The maximum likelihood estimation of linkage disequilibrium, *D*, is:

$D=\frac{1}{n}n_{A1B1}-2p_{A1}p_{B1}$ (1)

where *n* is the number of individuals and the digenic count, $n_{A1B1}$ , of *A_1_B_1_* + *A_1_*|*B_1_* is the following sum of genotype counts:

$n_{A1B1}= 2\left( A_{1}A_{1}B_{1}B_{1} \right)+ A_{1}A_{1}B_{1}B_{2}+A_{1}A_{2}B_{1}B_{1}+\frac{1}{2}(A_{1}A_{2}B_{1}B_{2})$ (1)

Using *D,* we estimated haplotype frequencies using the following equations:

$$A_{1}B_{1}=D+p_{A1}p_{B1}$$

$$A_{1}B_{2}=p_{A1}p_{B2}-D$$

$$A_{2}B_{1}=p_{A2}p_{B1}-D$$

$$A_{2}B_{2}=D+p_{A2}p_{B2}$$

References

1. Weir BS. Genetic Data Analysis II: Methods for Discrete Population Genetic Data: Sinauer Associates; 1996.
