# Supplementary material for: Fine-scale spatial and temporal dynamics of kdr haplotypes in Aedes aegypti from Mexico
Source: Parasit Vectors. 2019 Jan 9;12:20. doi: 10.1186/s13071-018-3275-9 (PMC6327429; doi:10.1186/s13071-018-3275-9)
Supplement: Supplementary file 2 — Table S1. Block-level frequencies of C1534 during all four sampling timepoints. Dry season collections were a combination of in-house adult sampling and adults emerging from ovitraps; sample size from each is indicated. Wet season collections only consisted of in-house adult samples. Significant differences are marked with asterisks after correcting for multiple comparisons. (DOCX 21 kb) [file 13071_2018_3275_MOESM2_ESM.docx]

**Additional file 2: Table S1.** Block-level frequencies of C1534 during all four sampling timepoints. Dry season collections were a combination of in-house adult sampling and adults emerging from ovitraps; sample size from each is indicated. Wet season collections only consisted of in-house adult samples. Significant differences are marked with asterisks after correcting for multiple comparisons.

| **C1534** | **Wet 2014** | | **Dry 2015** | | | **Wet 2015** | | **Dry 2016** | | |  |
| --- | --- | --- | --- | --- | --- | --- | --- | --- | --- | --- | --- |
| **Block** | **Freq** | **N** | **Freq** | **N house** | **N ovitrap** | **Freq** | **N** | **Freq** | **N house** | **N ovitrap** | **p-value** |
| **A** | 0.73 | 26 | 0.89 | 6 | 34 | 0.66 | 46 | 0.70 | 0 | 22 | 0.006 |
| **B** | 0.48 | 95 | 0.74 | 3 | 33 | 0.64 | 54 | 0.68 | 5 | 40 | <0.001* |
| **C** | 0.37 | 82 | 0.48 | 8 | 17 | 0.44 | 39 | 0.43 | 13 | 41 | 0.446 |
| **D** | 0.46 | 42 | 0.71 | 6 | 33 | 0.54 | 12 | 0.25 | 1 | 7 | 0.001* |
| **E** | 0.65 | 33 | 0.61 | 0 | 19 | 0.50 | 25 | 0.64 | 2 | 40 | 0.333 |
| **F** | 0.44 | 40 | 0.68 | 0 | 19 | 0.58 | 49 | 0.61 | 1 | 39 | 0.034 |
| **G** | 0.69 | 54 | 0.49 | 1 | 39 | 0.59 | 43 | 0.48 | 1 | 39 | 0.011 |
| **H** | 0.50 | 39 | 0.50 | 0 | 42 | 0.69 | 27 | 0.50 | 1 | 5 | 0.127 |
| **J** | 0.50 | 19 | 0.63 | 0 | 23 | 0.56 | 49 | 0.63 | 9 | 40 | 0.448 |
| **K** | 0.42 | 37 | 0.33 | 6 | 0 | 0.64 | 44 | 0.50 | 5 | 19 | 0.023 |
| **L** | 0.62 | 34 | 0.45 | 2 | 45 | 0.72 | 25 | 0.72 | 5 | 4 | 0.005 |
| **M** | 0.64 | 35 | 0.80 | 6 | 38 | 0.42 | 33 | 0.28 | 4 | 12 | <0.001* |
| **N** | 0.58 | 32 | 0.72 | 0 | 46 | 0.76 | 29 | 0.57 | 1 | 40 | 0.038 |
| **P** | 0.53 | 41 | 0.91 | 0 | 44 | 0.28 | 20 | 0.52 | 0 | 22 | <0.001* |
| **Q** | 0.50 | 28 | 0.69 | 8 | 0 | 0.82 | 11 | 0.88 | 0 | 24 | <0.001* |
| **R** | 0.47 | 43 | 0.70 | 9 | 49 | 0.48 | 21 | 0.82 | 2 | 36 | <0.001* |
| **S** | 0.43 | 37 | 0.52 | 1 | 50 | 0.71 | 12 | 0.67 | 0 | 39 | 0.011 |
| **T** | 0.42 | 49 | 0.44 | 2 | 30 | 0.33 | 35 | 0.78 | 3 | 29 | <0.001* |
| **U** | 0.54 | 52 | 0.77 | 0 | 28 | 0.61 | 18 | 0.82 | 0 | 20 | 0.002* |
| **V** | 0.57 | 54 | 0.68 | 15 | 13 | 0.64 | 18 | 0.50 | 1 | 14 | 0.328 |
| **W** | 0.49 | 51 | -- | 0 | 0 | 0.52 | 30 | 0.67 | 1 | 40 | 0.038 |
| **X** | 0.54 | 56 | 0.93 | 1 | 29 | 0.83 | 15 | -- | 0 | 0 | <0.001* |
| **Y** | 0.54 | 61 | 0.65 | 8 | 22 | 0.78 | 20 | 0.90 | 0 | 31 | <0.001* |
| **Z** | 0.53 | 30 | 0.83 | 16 | 13 | 0.64 | 33 | -- | 0 | 0 | 0.001* |
| ***p*-value** | <0.001* |  | <0.001* |  |  | <0.001* |  | <0.001* |  |  |  |
