# Supplementary material for: Fine-scale spatial and temporal dynamics of kdr haplotypes in Aedes aegypti from Mexico
Source: Parasit Vectors. 2019 Jan 9;12:20. doi: 10.1186/s13071-018-3275-9 (PMC6327429; doi:10.1186/s13071-018-3275-9)
Supplement: Supplementary file 3 — Table S2. Block-level frequencies of I1016 during all four sampling timepoints. Dry season collections were a combination of in-house adult sampling and adults emerging from ovitraps; sample size from each is indicated. Wet season collections only consisted of in-house adult samples. Significant differences are marked with asterisks after correcting for multiple comparisons. (DOCX 21 kb) [file 13071_2018_3275_MOESM3_ESM.docx]

**Additional file 3: Table S2.** Block-level frequencies of I1016 during all four sampling timepoints. Dry season collections were a combination of in-house adult sampling and adults emerging from ovitraps; sample size from each is indicated. Wet season collections only consisted of in-house adult samples. Significant differences are marked with asterisks after correcting for multiple comparisons.

| **I1016** | **Wet 2014** | | **Dry 2015** | | | **Wet 2015** | | **Dry 2016** | | |  |
| --- | --- | --- | --- | --- | --- | --- | --- | --- | --- | --- | --- |
| **Block** | **Freq** | **N** | **Freq** | **N house** | **N ovitrap** | **Freq** | **N** | **Freq** | **N house** | **N ovitrap** | **p-value** |
| **A** | 0.60 | 25 | 0.63 | 7 | 36 | 0.40 | 46 | 0.69 | 0 | 21 | 0.003 |
| **B** | 0.18 | 22 | 0.38 | 3 | 31 | 0.44 | 51 | 0.55 | 4 | 39 | <0.001* |
| **C** | 0.30 | 27 | 0.38 | 7 | 17 | 0.38 | 37 | 0.30 | 13 | 41 | 0.562 |
| **D** | 0.43 | 22 | 0.55 | 3 | 34 | 0.46 | 12 | 0.12 | 1 | 7 | 0.019 |
| **E** | 0.64 | 11 | 0.74 | 0 | 25 | 0.54 | 23 | 0.54 | 2 | 40 | 0.100 |
| **F** | 0.35 | 27 | 0.67 | 0 | 6 | 0.38 | 46 | 0.40 | 1 | 40 | 0.238 |
| **G** | 0.50 | 53 | 0.44 | 1 | 31 | 0.31 | 42 | 0.40 | 1 | 39 | 0.066 |
| **H** | 0.45 | 39 | 0.19 | 0 | 37 | 0.42 | 24 | 0.17 | 1 | 5 | 0.002* |
| **J** | 0.45 | 19 | 0.52 | 0 | 27 | 0.41 | 48 | 0.36 | 9 | 40 | 0.270 |
| **K** | 0.26 | 37 | 0.14 | 7 | 0 | 0.46 | 41 | 0.24 | 5 | 20 | 0.006 |
| **L** | 0.43 | 34 | 0.29 | 3 | 49 | 0.50 | 25 | 0.39 | 5 | 4 | 0.060 |
| **M** | 0.61 | 33 | 0.67 | 6 | 41 | 0.26 | 33 | 0.13 | 3 | 12 | <0.001* |
| **N** | 0.45 | 32 | 0.61 | 0 | 47 | 0.45 | 22 | 0.34 | 1 | 37 | 0.007 |
| **P** | 0.52 | 41 | 0.78 | 0 | 47 | 0.38 | 13 | 0.37 | 0 | 19 | 0.001* |
| **Q** | 0.45 | 28 | 0.64 | 7 | 0 | 0.61 | 9 | 0.58 | 0 | 24 | 0.349 |
| **R** | 0.40 | 42 | 0.53 | 8 | 50 | 0.45 | 20 | 0.64 | 2 | 36 | 0.019 |
| **S** | 0.41 | 37 | 0.60 | 1 | 30 | 0.50 | 12 | 0.50 | 0 | 39 | 0.175 |
| **T** | 0.39 | 49 | 0.33 | 2 | 30 | 0.40 | 34 | 0.61 | 3 | 29 | 0.007 |
| **U** | 0.48 | 52 | 0.57 | 0 | 30 | 0.53 | 15 | 0.15 | 0 | 20 | <0.001* |
| **V** | 0.41 | 54 | 0.43 | 16 | 14 | 0.50 | 17 | 0.23 | 1 | 14 | 0.162 |
| **W** | 0.29 | 51 | -- | 0 | 0 | 0.40 | 31 | 0.30 | 1 | 40 | 0.313 |
| **X** | 0.40 | 56 | 0.57 | 1 | 29 | 0.62 | 16 | -- | 0 | 0 | 0.027 |
| **Y** | 0.43 | 61 | 0.50 | 8 | 22 | 0.55 | 19 | 0.47 | 0 | 31 | 0.600 |
| **Z** | 0.38 | 30 | 0.67 | 17 | 13 | 0.59 | 29 | -- | 0 | 0 | 0.006 |
| ***p*-value** | <0.001* |  | <0.001* |  | | 0.032* |  | <0.001* |  |  |  |
