# Supplementary material for: Fine-scale spatial and temporal dynamics of kdr haplotypes in Aedes aegypti from Mexico
Source: Parasit Vectors. 2019 Jan 9;12:20. doi: 10.1186/s13071-018-3275-9 (PMC6327429; doi:10.1186/s13071-018-3275-9)
Supplement: Supplementary file 4 — Table S3. Haplotype frequencies for each block at each sampling timepoint. (DOCX 25 kb) [file 13071_2018_3275_MOESM4_ESM.docx]

**Additional file 4: Table S3**. Haplotype frequencies for each block at each sampling timepoint.

| **Wet season 2014** | | | | | |
| --- | --- | --- | --- | --- | --- |
| **Block** | **N** | **C1534/I1016** | **C1534/V1016** | **F1534/I1016** | **F1534/V1016** |
| A | 22 | 0.574 | 0.176 | 0 | 0.256 |
| B | 22 | 0.155 | 0.118 | 0.027 | 0.7 |
| C | 30 | 0.227 | 0.206 | 0.056 | 0.511 |
| D | 22 | 0.436 | 0.132 | 0 | 0.436 |
| E | 8 | 0.641 | 0.234 | 0 | 0.141 |
| F | 27 | 0.337 | 0.126 | 0.015 | 0.522 |
| G | 53 | 0.509 | 0.189 | 0 | 0.311 |
| H | 2 | -- | -- | -- | -- |
| J | 19 | 0.382 | 0.118 | 0.066 | 0.434 |
| K | 37 | 0.217 | 0.202 | 0.04 | 0.541 |
| L | 34 | 0.384 | 0.234 | 0.043 | 0.34 |
| M | 32 | 0.59 | 0.066 | 0.035 | 0.309 |
| N | 32 | 0.426 | 0.153 | 0.028 | 0.394 |
| P | 41 | 0.481 | 0.068 | 0.044 | 0.407 |
| Q | 28 | 0.491 | 0.009 | 0 | 0.545 |
| R | 42 | 0.407 | 0.057 | 0 | 0.538 |
| S | 37 | 0.446 | 0 | 0 | 0.608 |
| T | 49 | 0.419 | 0 | 0 | 0.613 |
| U | 52 | 0.472 | 0.067 | 0.009 | 0.453 |
| V | 54 | 0.4 | 0.165 | 0.008 | 0.427 |
| W | 51 | 0.277 | 0.213 | 0.017 | 0.493 |
| X | 56 | 0.37 | 0.174 | 0.031 | 0.424 |
| Y | 61 | 0.437 | 0.104 | 0 | 0.462 |
| Z | 30 | 0.352 | 0.165 | 0.031 | 0.452 |
| **Dry season 2015** | | | | | |
| **Block** | **N** | **C1534/I1016** | **C1534/V1016** | **F1534/I1016** | **F1534/V1016** |
| A | 40 | 0.645 | 0.242 | 0 | 0.133 |
| B | 34 | 0.416 | 0.305 | 0 | 0.313 |
| C | 23 | 0.459 | 0.062 | 0 | 0.568 |
| D | 35 | 0.627 | 0.088 | 0 | 0.369 |
| E | 18 | 0.594 | 0.045 | 0.128 | 0.233 |
| F | 6 | 0.722 | 0 | 0 | 0.389 |
| G | 32 | 0.393 | 0.139 | 0.045 | 0.424 |
| H | 36 | 0.258 | 0.269 | 0 | 0.536 |
| J | 20 | 0.56 | 0.09 | 0.04 | 0.31 |
| K | 6 | 0.056 | 0.278 | 0.028 | 0.639 |
| L | 46 | 0.307 | 0.128 | 0 | 0.579 |
| M | 44 | 0.632 | 0.163 | 0.015 | 0.189 |
| N | 45 | 0.713 | 0 | 0 | 0.38 |
| P | 44 | 0.775 | 0.134 | 0 | 0.093 |
| Q | 7 | 0.709 | 0.077 | 0 | 0.281 |
| R | 57 | 0.492 | 0.201 | 0.025 | 0.282 |
| S | 31 | 0.779 | 0 | 0 | 0.57 |
| T | 32 | 0.325 | 0.112 | 0.003 | 0.56 |
| U | 28 | 0.677 | 0.091 | 0 | 0.302 |
| V | 28 | 0.423 | 0.255 | 0.005 | 0.316 |
| W | 0 |  |  |  |  |
| X | 30 | 0.538 | 0.396 | 0.029 | 0.038 |
| Y | 30 | 0.442 | 0.208 | 0.058 | 0.292 |
| Z | 29 | 0.734 | 0.094 | 0 | 0.251 |
| **Wet season 2015** | | | | | |
| **Block** | **N** | **C1534/I1016** | **C1534/V1016** | **F1534/I1016** | **F1534/V1016** |
| A | 46 | 0.386 | 0.278 | 0.017 | 0.32 |
| B | 50 | 0.477 | 0.154 | 0 | 0.397 |
| C | 37 | 0.34 | 0.12 | 0.039 | 0.502 |
| D | 11 | 0.413 | 0.132 | 0 | 0.459 |
| E | 22 | 0.545 | 0 | 0 | 0.499 |
| F | 46 | 0.414 | 0.184 | 0 | 0.436 |
| G | 42 | 0.336 | 0.271 | 0 | 0.419 |
| H | 24 | 0.398 | 0.248 | 0.019 | 0.335 |
| J | 48 | 0.359 | 0.193 | 0.047 | 0.401 |
| K | 40 | 0.424 | 0.226 | 0.038 | 0.312 |
| L | 23 | 0.467 | 0.25 | 0.033 | 0.25 |
| M | 32 | 0.29 | 0.147 | 0 | 0.587 |
| N | 22 | 0.469 | 0.349 | 0 | 0.196 |
| P | 13 | 0.228 | 0.08 | 0.157 | 0.536 |
| Q | 8 | 0.578 | 0.172 | 0 | 0.266 |
| R | 20 | 0.4 | 0.1 | 0.05 | 0.45 |
| S | 12 | 0.479 | 0.229 | 0.021 | 0.271 |
| T | 34 | 0.23 | 0.079 | 0.167 | 0.524 |
| U | 15 | 0.511 | 0.156 | 0.022 | 0.311 |
| V | 16 | 0.457 | 0.168 | 0.012 | 0.363 |
| W | 30 | 0.327 | 0.19 | 0.073 | 0.41 |
| X | 14 | 0.608 | 0.213 | 0 | 0.18 |
| Y | 19 | 0.497 | 0.319 | 0.056 | 0.128 |
| Z | 26 | 0.528 | 0.145 | 0.029 | 0.298 |
| **Dry season 2016** | | | | | |
| **Block** | **N** | **C1534/I1016** | **C1534/V1016** | **F1534/I1016** | **F1534/V1016** |
| A | 21 | 0.626 | 0.088 | 0.065 | 0.221 |
| B | 43 | 0.526 | 0.172 | 0.021 | 0.282 |
| C | 54 | 0.328 | 0.098 | 0 | 0.605 |
| D | 8 | 0.094 | 0.156 | 0.031 | 0.719 |
| E | 42 | 0.584 | 0.059 | 0 | 0.406 |
| F | 40 | 0.425 | 0.187 | 0 | 0.425 |
| G | 39 | 0.364 | 0.123 | 0.046 | 0.467 |
| H | 6 | 0.167 | 0.333 | 0 | 0.5 |
| J | 49 | 0.315 | 0.318 | 0.042 | 0.325 |
| K | 24 | 0.323 | 0.177 | 0 | 0.594 |
| L | 9 | 0.497 | 0.225 | 0 | 0.386 |
| M | 15 | 0.193 | 0.107 | 0 | 0.76 |
| N | 38 | 0.443 | 0.11 | 0 | 0.548 |
| P | 19 | 0.421 | 0.079 | 0 | 0.553 |
| Q | 24 | 0.594 | 0.281 | 0 | 0.135 |
| R | 38 | 0.606 | 0.21 | 0.039 | 0.145 |
| S | 38 | 0.511 | 0.16 | 0.002 | 0.327 |
| T | 32 | 0.727 | 0.054 | 0 | 0.336 |
| U | 20 | 0.126 | 0.699 | 0.024 | 0.151 |
| V | 15 | 0.217 | 0.283 | 0.017 | 0.483 |
| W | 41 | 0.332 | 0.339 | 0 | 0.356 |
| X | 0 |  |  |  |  |
| Y | 31 | 0.448 | 0.455 | 0.019 | 0.078 |
| Z | 2 | -- | -- | -- | -- |
