# Supplementary material for: Fine-scale spatial and temporal dynamics of kdr haplotypes in Aedes aegypti from Mexico
Source: Parasit Vectors. 2019 Jan 9;12:20. doi: 10.1186/s13071-018-3275-9 (PMC6327429; doi:10.1186/s13071-018-3275-9)
Supplement: Supplementary file 5 — Table S4. Haplotype frequencies for each sector at each sampling timepoint. (DOCX 14 kb) [file 13071_2018_3275_MOESM5_ESM.docx]

**Additional file 5: Table S4.** Haplotype frequencies for each sector at each sampling timepoint.

| **Wet season 2014** | | | | | |
| --- | --- | --- | --- | --- | --- |
| **Sector** | **N** | **C1534/I1016** | **C1534/V1016** | **F1534/I1016** | **F1534/V1016** |
| 1 | 395 | 0.418 | 0.127 | 0.009 | 0.445 |
| 2 | 136 | 0.418 | 0.137 | 0 | 0.452 |
| 3 | 152 | 0.368 | 0.159 | 0.034 | 0.44 |
| 4 | 158 | 0.441 | 0.037 | 0 | 0.549 |
| **Dry season 2015** | | | | | |
| 1 | 236 | 0.628 | 0.19 | 0 | 0.202 |
| 2 | 153 | 0.483 | 0.148 | 0 | 0.421 |
| 3 | 139 | 0.488 | 0.102 | 0 | 0.445 |
| 4 | 173 | 0.558 | 0.072 | 0 | 0.405 |
| **Wet season 2015** | | | | | |
| 1 | 187 | 0.445 | 0.2 | 0.031 | 0.324 |
| 2 | 184 | 0.419 | 0.222 | 0 | 0.381 |
| 3 | 180 | 0.378 | 0.178 | 0.022 | 0.422 |
| 4 | 99 | 0.39 | 0.08 | 0.06 | 0.471 |
| **Dry season 2016** | | | | | |
| 1 | 173 | 0.41 | 0.319 | 0 | 0.28 |
| 2 | 166 | 0.437 | 0.151 | 0 | 0.434 |
| 3 | 151 | 0.327 | 0.183 | 0 | 0.522 |
| 4 | 158 | 0.59 | 0.11 | 0 | 0.34 |
